# Can Nuts Mitigate Malnutrition in Older Adults? A Conceptual Framework

**DOI:** 10.3390/nu10101448

**Published:** 2018-10-06

**Authors:** Sze-Yen Tan, Siew Ling Tey, Rachel Brown

**Affiliations:** 1 Institute for Physical Activity and Nutrition (IPAN), School of Exercise and Nutrition Sciences, Deakin University, Geelong, VIC 3220, Australia; 2 Department of Human Nutrition, University of Otago, PO Box 56, Dunedin 9054, New Zealand; siewling.tey@otago.ac.nz (S.L.T.); rachel.brown@otago.ac.nz (R.B.)

**Keywords:** Nuts, ageing, nutritional status, malnutrition, appetite, energy intake, health

## Abstract

The proportion of adults aged over 60 years in the world is expected to reach 20% by the year 2050. Ageing is associated with several physiological changes that increase the risk of malnutrition among this population. Malnutrition is characterized by deficiencies or insufficiencies of macro- and micronutrients. Malnutrition has detrimental effects on the health, wellbeing, and quality of life in older adults. Nuts are rich in energy, unsaturated fats, and protein, as well as other nutrients that provide a range of health benefits. While the effects of nuts on overnutrition have been studied extensively, very few studies have been specifically designed to understand the role of nuts in mitigating undernutrition in the elderly. Therefore, this review explores the potential role of nuts in improving the nutritional status of older adults who are at risk of undernutrition. Several properties of whole nuts, some of which appear important for addressing overnutrition, (e.g., hardness, lower-than-expected nutrient availability, satiety-enhancing effects) may limit their effectiveness as a food to combat undernutrition. However, we propose that modifications such as transforming the physical form of nuts, addressing the timing of nut ingestion, and introducing variety may overcome these barriers. This review also discusses the feasibility of using nuts to prevent and reverse undernutrition among older adults. We conclude with a recommendation to conduct clinical studies in the future to test this conceptual framework.

## 1. Introduction: Ageing and Risks of Malnutrition

Based on the United Nations Department of Economic and Social Affairs’ report ‘World Population Ageing’ released in 2015, approximately 12.5% of the world population is aged 60 years or over [1]. This number is projected to reach 20% by 2050, and this population will overtake adolescent and youth groups. The ageing population will have a big impact on several aspects, from economic growth to increasing needs for medical care and aged-care facilities.

Ageing is the cause of several health concerns. Older adults face: (1) frailty and mobility difficulties due to arthritis and osteoporosis, (2) a proneness to falls due to sarcopenia, (3) chronic diseases such as obesity, cardiovascular disease, type 2 diabetes, and cancer, (4) decreasing impairments in mental health such as Alzheimer’s disease, depression, and dementia, and (5) poor eyesight and dentition [2]. In addition, some older adults experience decreased appetite and/or food intake [3,4]. All these health issues, combined with loss of income and living in isolation, influence older adults’ ability to shop for groceries, prepare foods, and eat sufficiently to support healthy ageing. Poor dietary intake, coupled with increased nutritional requirements due to poor health, may lead to malnutrition. Malnutrition is a common nutritional issue faced by older adults, and can worsen their health conditions and impact on their quality of life. Malnutrition is associated with higher healthcare costs in community-dwelling elderly and institutionalized elderly [5,6,7].

To make matters worse, malnutrition may co-exist with other conditions such as being overweight [8], representing a double burden among older adults [9]. Malnutrition is prevalent globally in both developing and developed countries and is ubiquitous across hospitals, long-term care facilities, and community settings. A cross-sectional study conducted in Australia reported that 17% of elderly aged 75 years and above were at risk of malnutrition, in which 34% of were overweight (body mass index (BMI) defined as the ratio of weight in kg to (height in meter)^2^ ≥ 25 kg/m^2^) and a further 13% were underweight (BMI < 18.5 kg/m^2^) [10]. This finding highlights that a double burden of malnutrition exists, and overweight and obese elderly are equally vulnerable to malnutrition as underweight elderly [10].

A recent meta-analysis reported the prevalence of malnutrition and risk of malnutrition using the full version of the Mini Nutritional Assessment tool in 113,967 older adults aged 60 years and above from 240 studies [11]. The prevalence of malnutrition ranged from 3.1% in community-dwelling elderly to 29% among elderly in long-term care, rehabilitation, and sub-acute care. A similar trend was observed for the prevalence of malnutrition risk, whereby 26.5% of the community-dwelling elderly and 49% of the elderly in rehabilitation and sub-acute care were identified as at risk of malnutrition [11].

The aetiology of malnutrition is multifactorial and is likely due to physiological, psychological, environmental, and sociological changes which accompany the ageing process. In addition, older adults are at greater risk of dietary insufficiency due to age-related increases in nutrient requirements concomitant with a reduction in energy requirements and decreases in appetite and energy intake [4,12]. Compromised chewing and swallowing may also affect food intake in the elderly [13,14,15]. Due to suboptimal food intake among the elderly, they are at increased risk for insufficient energy and protein intake. Micronutrient insufficiencies that are common in the elderly include vitamin D, vitamin E, vitamin B6, vitamin B12, folate, calcium, magnesium, iron, and zinc deficiencies [12,16,17].

Given that undernutrition impacts on several aspects of older adults’ quality of life, functionality, and health status, finding effective dietary strategies to combat this condition is of utmost importance. The World Health Organization [18] emphasizes the importance of supplementation which is high in energy, protein, vitamins, and minerals for older adults who are undernourished. Nuts have high energy density and are rich sources of unsaturated fatty acids, protein, dietary fibre, phytochemicals, and micronutrients [19,20]. In this review, we explore and propose the use of nuts in enhancing the nutritional intake of older adults based on available knowledge on the health effects of nuts. We use the term ‘undernutrition’ from this point onwards to describe suboptimal nutritional intake, which represents an early stage that leads to malnutrition if not corrected.

## 2. Nuts, Energy Balance and Undernutrition: Opportunities and Challenges

Nuts are nutrient-rich, and research on how nuts influence human energy balance has largely focused on weight gain prevention and weight loss promotion in overweight and obese populations [21]. However, studies on the effects of nuts on improving the nutritional status of older adults are limited. Since both under- and overnutrition represent two extremes of the nutritional status spectrum, vast knowledge on how nuts could assist obesity management accumulated thus far is arguably translatable to reversing undernutrition. Fundamentally, body weight is determined by the cumulative effects of energy balance over a period of time. From a thermodynamic viewpoint, energy excess (energy intake > energy expenditure) promotes weight gain, while an energy deficit (energy intake < energy expenditure) results in weight loss. Given the scope of this review on the potential role of nuts in improving the nutrition status of older adults, the role of energy expenditure is acknowledged but will not be discussed extensively.

**Figure 1:**
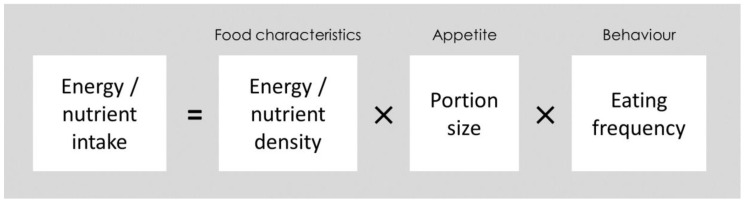
A conceptual framework of how nuts may increase energy/nutrient intake by influencing energy/nutrient density, portion size, and eating behaviours, and subsequently aid in the reversal/prevention of undernutrition.

Increasing total energy intake is a priority in the prevention and management of undernutrition. When overall energy consumption is increased, the intake of other important nutrients pertinent to undernutrition is likely to be elevated as well. This is likely to be more pronounced when foods which are nutrient-dense are added to the diet. In this review, we will articulate how nuts can address all these factors concurrently. The conceptual framework used as an outline to support our argument that nuts are ideal candidates to prevent and reverse undernutrition is shown in Figure 1 below. In this conceptual framework, we propose that energy and nutrient intake is determined by three major factors i.e., energy and nutrient density, the portion size of foods ingested, and the frequency of eating. In the context of undernutrition, energy intake can be increased by frequent consumption of highly energy-dense foods, in greater portion sizes. However, from a nutrition perspective, foods should not be viewed as energy per se. Therefore, food intake is also driven by several other factors such as food tolerance, preference or liking towards a food, palatability of a food, and food variety. These factors are highly relevant to the elderly population and will also be elaborated in this review, using nuts as an example.

### 2.1. Food Characteristics: Energy Density

Observational studies have reported that higher intake of energy-dense foods was associated with the energy intake of both children and adults [22,23]. In addition, research has suggested that humans often guide food intake visually via portion sizes instead of the energy density of a particular food or meal [24]. This effect appears particularly true during the ingestion of unfamiliar foods. Experimentally, the manipulation of energy density of test meals, while maintaining the portion size, led to significant changes in energy intake from that meal, which subsequently extended to the total daily energy intake [25]. This observation is not too surprising because energy densities of foods or beverages are less easily judged by consumers than their portion sizes. The effects of higher energy density foods on energy intake are often long-lasting and translate into higher body weight in the population. Spontaneous adjustment to food intake in response to energy density manipulation has been reported but this appears to occur mostly when the energy density of a test meal is reduced [26].

Based on observational studies, the energy density of foods is associated with energy intake and it presents a major challenge to individuals who intend to maintain or lose weight [22]. The higher energy density of some foods can be explained by the higher carbohydrate (or sugar) and fat content in the food [27], which is also partly attributed to food processing. While the situation may look dire for weight management, increasing energy and nutrient density via food processing and fortification presents an opportunity to prevent and reverse undernutrition. The most apparent examples can be drawn from food fortification strategies that combat micronutrient deficiencies [28]. In the context of undernutrition among the elderly, recent reviews suggest that the fortification of meals and snack provision are effective in increasing the energy and protein intake of older adults in community, long-term care, and hospital settings [29,30]. An alternative strategy to food processing and fortification is the selection of foods that are naturally high in energy such as nuts. Apart from their high energy content (approximately 29 kJ/g or 7 kcal/g), nuts are also high in micronutrients, and how these nutrients improve diet quality will be discussed in greater detail in Section 4. As highlighted earlier, ageing is a risk factor for several diseases and the unique nutrient profiles of nuts have been shown to be beneficial to older adults. For example, nuts are protective against metabolic diseases [31], and promote vascular health and mental health [32]. Nuts are a good source of protein, which may help preserve lean mass and motor function of older adults.

The use of nuts to improve nutritional status is not a new idea. In fact, peanut butter fortified with calcium and iron (a ready-to-use therapeutic food known as Plumpy’nut) has been used to fight malnutrition among children and pregnant women in several countries in Africa and Asia [33,34,35]. Based on the success of Plumpy’nut, we propose that nuts, which are energy- and nutrient-dense, are also suitable to prevent or reverse undernutrition among older adults.

### 2.2. Appetite Regulation: Portion Size

Portion size is determined by a number of factors. When processed and convenience foods are consumed, portion size is largely influenced by packaging size. However, in a buffet setting where foods and beverages are not limited, the amount of foods consumed is guided visually [24] and governed by appetite [36] despite some misgivings of these regulatory systems [37]. During weight loss, individuals are advised to select foods that are highly satiating in order to reduce food intake. Foods that provide greater satiation and satiety are in a solid form [38], consumed in bigger portion sizes [39], and are high in certain nutrients such as dietary protein [40], and fibre [41]. Nuts have long been recognised as an ideal food to support weight management because of their attributes above that regulate energy intake. Whole nuts require a significant amount of mastication, and longer oral processing and sensory exposure time has been shown to promote fullness [42]. Because nuts are filling, the ingestion of nuts may displace the amount of foods consumed at a meal. Studies have shown that 54–151% of energy from nuts is compensated via a spontaneous reduction in subsequent food intake [43,44,45,46]. Based on the evidence, the appetitive effects of nuts may seem to favour overweight and obese older adults rather than those at risk for undernutrition. To further complicate the matter, older adults may suffer from loss of appetite, further limiting food intake and making them susceptible to undernutrition. However, some modifications to nuts may overcome these limitations and make them appropriate in the context of undernutrition. These strategies are proposed and described in detail in the next section.

## 3. Strategies to Incorporate Nuts into the Habitual Diets of Older Adults

In order for nuts to be suitable in the prevention and reversal of undernutrition among older adults, strategies such as the modification of nut forms, introduction of variety to minimise the known appetitive effects of nuts, and timing of nut intake may be appropriate. These strategies can be used to guide the development of nut products that are suitable, convenient, and affordable to improve the nutritional status of older adults.

### 3.1. Nut Forms

Although nuts are naturally high in energy and essential nutrients, these nutrients are not readily accessible to humans. Microscopic analysis of nuts revealed that the unsaturated fats of nuts are encapsulated within thick cell walls, hence limiting their availability [47]. Indeed, human studies reported that only 68% of energy from almonds [48], 95% from pistachios [49], and 79% of energy from walnuts [50] were available and absorbed by the human body.

To address the issues of lower nutrient accessibility from nuts, studies have demonstrated that oral mastication [51,52] and food processing [53] increase the nutrient release from nuts. Food processing (e.g., roasting) alters the hardness of nuts and allows roasted nuts to fracture into smaller particles, hence releasing more of the fats in nuts for absorption [53,54]. Further processing of nuts into a butter form [55,56] or into pure oil [57] leads to higher nutrient absorption. Findings from these studies provide important information on how the manipulation of the physical form of nuts may make them effective in managing undernutrition. Therefore, nut butter appears to be a viable form of nutrient delivery. Preferably, nut butter should be prepared via food processing instead of through mastication, in order to minimise its appetitive effects due to oro-sensory exposure [42]. In addition, many older adults may suffer from dentition issues and thus have problems with mastication. The benefits of nuts in preventing and reversing undernutrition could be further maximised if nut butters are added during milkshake preparation with dairy or non-dairy-base such as e.g., nut and rice milks. Not only will adding nut butter to milk increase the nutrient density of this beverage, it is suitable as a between-meal snack or to replace water when older adults take their medications (a strategy commonly used in acute care settings). As highlighted above, beverages are less satiating than solid foods. More importantly, beverages can be consumed frequently throughout the day as opposed to solid foods, which are often consumed as three main meals and snacks. The use of oral nutrition supplement has been shown to improve nutritional status of older adults in an aged-care facility setting [58] and this strategy has the potential to be equally effective in a free-living environment [59]. This idea is further supported by a systematic review, which concluded that there is high compliance to high-energy oral nutritional supplements and they have been shown to overcome poor oral food intake [60]. Besides nutritional intake, the use of nut butters or milkshakes may have other benefits. As highlighted earlier, poor dentition is a common barrier to nut intake in older adults [61], and whole nuts may be a choking risk for the elderly. Therefore, delivering nutrient in butter or beverage form will overcome the common nutritional issues faced by older adults.

### 3.2. Variety

In conjunction with increasing the nutrient availability of nuts via physical form modification, it is equally important to consider ways to enhance the compliance to nut consumption among the elderly. Apart from flavour and costs, another common reason of non-compliance to dietary supplements reported in the literature is sensory-specific satiety due to monotony. Sensory-specific satiety describes a condition where the repeated consumption of a same food due to limited choices leads to a decrease in the liking to that food and subsequently reduces food intake [62]. This is a considerable problem, especially among older adults at risk for undernutrition. Research in the area of sensory-specific satiety suggests that increasing the variety of foods may reverse this condition. Therefore, to promote compliance to nut consumption among older adults, a variety of nuts in various forms and flavours should be considered. From a nutrition perspective, most nuts have comparable macronutrient profiles, with one exception being walnuts, which are high in polyunsaturated fats and may have additional benefits on heart health and cognition. Also, although all nuts are rich sources of micronutrients, different nuts types differ in their nutrient profiles, meaning increasing variety will improve the intakes of a wider range of nutrients. The various forms of nuts may also be suitable options for older adults with no dentition problems. Previous studies have shown that whole, sliced, chopped nuts and flavoured nuts are liked and there is no evidence of a difference in the health benefits of the different forms and flavours [63,64,65,66]. In other words, various types of nuts in various forms should be used to improve the nutritional status and the compliance of older adults to regular nut consumption.

### 3.3. Timing

The timing of nut consumption is also a crucial consideration. If nuts are consumed together with a meal, it is likely that the satiating effects of nuts will reduce the intake of other foods in the same meal, hence creating an undesirable ‘food displacement’ effect. When loss of appetite occurs, older adults may be able to tolerate smaller meals and small-and-frequent meal patterns may be more desirable to ensure sufficient food intake. For this reason, it is recommended that nuts should be ingested as snacks, rather than together with a meal to avoid a reduction in main meal intake. This idea is supported by several studies where snacks have been reported to be important contributors of daily essential nutrient intake in both children and adults [67,68]. Snacking has also been shown to increase the intake of vitamins and minerals in older adults [69]. The timing of nut intake could be coupled with the manipulation of nut forms (e.g., nut butter or nut milkshake) in order to maximise their effects on preventing or reversing undernutrition in the elderly.

## 4. Improving Diet Quality with Nuts

Although individual nut types differ in nutrient composition, they are all considered to be nutrient-dense. This nutrient profile is likely to contribute to their well-recognised health properties. Nuts are typically rich sources of cis-unsaturated fatty acids, fibre, and plant protein [20,70]. Individual nut types contribute useful amounts of vitamin E, folate, calcium, copper, iron, magnesium, phosphorous, potassium, and zinc. For example, a 30 g serving of almonds can provide over 100% of the daily value of vitamin E among older adults, whereas a serving of cashew nuts can provide nearly three-quarters of the daily value of copper and 25% of the daily value of iron. One serving of Brazil nuts contains up to 10-fold the daily value for selenium and around a third of the daily value of magnesium and phosphorous. A serving of pistachios provides over 10% of the daily value for potassium, and pine nuts contain nearly one-third of the daily value for zinc (Table 1).

A number of epidemiological studies have shown that diet quality is better among nut consumers compared to non-nut consumers [72,73,74,75,76]. For example, an analysis of the National Health and Nutrition Examination Survey (NHANES) 2005–2010 cohort showed that nut consumers were less likely to have inadequate intakes of vitamins A, C, E, folate, calcium, iron, magnesium, and zinc compared to non-nut consumers [76]. This study also reported that among nut consumers, nuts provided around 10% of total energy (TE), around 20% of total fat intake, and 10% of protein intake.

In support of this epidemiological research, intervention studies report improved diet quality when nuts are added to the diet [77,78,79,80], in a dose-response manner [81]. One intervention study carried out in an elderly population reported that the addition of walnuts to the usual diet (15% of energy, around 43 g) resulted in significantly higher intakes of protein, polyunsaturated fat, and a number of micronutrients, including magnesium, manganese and copper compared to those who did not consume walnuts [78]. Conversely, those who consumed walnuts had significantly lower intakes of carbohydrate, saturated fat and sodium than those who did not consume walnuts.

Collectively, this research suggests that regular consumption of nuts, which are nutrient-dense, is likely to improve diet quality. This will be useful for the elderly, where reductions in total food intake as a result of altered hunger and satiety sensations, can lead to inadequate intakes of protein and important micronutrients. Micronutrient insufficiencies that are common in the elderly include vitamin D, vitamin E, vitamin B6, folate, calcium, magnesium, iron, and zinc [12,16,17]. For instance, almonds and hazelnuts are rich sources of vitamin E, peanuts and hazelnuts are high in folate, Brazil nuts are high in magnesium selenium, pine nuts are high in zinc, cashew nuts are rich in iron, and pistachios contain reasonable amounts of vitamin B6. Therefore, the consumption of a variety of nuts can be used as a means to help elderly to meet the nutrient intake that are often fall short in their diet.

## 5. Overcoming Potential Barriers and Side-Effects of Nuts

### 5.1. Phytate

As noted above, nuts are nutrient-dense, containing useful amounts of fatty acids, vitamins, minerals, and a number of phytonutrients. However, nuts also contain appreciable amounts of phytate, an inhibitory compound which binds with minerals, forming complexes which interfere with the absorption of these nutrients [82,83,84,85]. This is not likely to problematic among those following an omnivorous diet which contains minerals from a number of food sources. High phytate levels in the diet are likely to be more of a concern among those where food intake may be compromised, such as in the elderly, and the nutrient density of the diet is poor. This is likely to be exacerbated amongst those following vegetarian and vegan diets where phytate concentrations are likely to be high and mineral availability is lower. 

One solution reported widely in the lay literature is activating (or soaking) nuts in order to reduce the phytate content and enhance mineral bioavailability. While this has proven a useful method in developing countries where it has been shown that the phytate content of legumes and grains, which are often staples, is lowered upon soaking, especially when they are mechanically broken down [86,87,88], little research has investigated the effects of soaking on the phytate and mineral content of nuts. To the best of our knowledge, two published studies have analysed the phytate content of soaked nuts [89,90]. Both studies found that soaking whole nuts increased phytate concentrations compared to untreated nuts. This could be due to the combination of no or minimal loss of phytate during the soaking process, and loss of moisture during the drying period, as nuts were dried following soaking, which subsequently resulted in a higher concentration of phytate in soaked or dried whole nuts. These results do not support claims that soaking nuts decreases the phytate content of nuts. As such, this process perhaps does not confer additional benefits to older adults. 

### 5.2. Dental Issues

Dentition issues have been reported as a barrier to regular nut consumption. This problem is likely to be more prevalent among the elderly. A recent survey which examined barriers to and facilitators of nut consumption among the general public in New Zealand found that dental issues was the most frequently reported reason for the avoidance of nut consumption [91]. In addition, in a survey among health professionals (dietitians, general practitioners, practice nurses), dental issues were reported by around 14% of participants as a reason why they advise some of their patients to eat fewer nuts [92]. Furthermore, among dietitians, dental issues were one of the top five reasons why they advised their patients to eat fewer nuts. Given these reported issues, different forms of nuts may be more suitable for those with dentition issues, such as older adults. Nut butters, and potentially sliced nuts, are obvious options, however, more information is needed on the acceptance of various nut form among the elderly.

### 5.3. Allergies and Aflatoxins

Nut allergies are one of the most common food allergies, and allergens and aflatoxins in nuts are two major concerns of consumers [93,94]. Most nuts contain the same allergens and thus, individuals who are allergic to one nut type may have a high level of co-allergy (cross-reactivity) to other closely related nut species [95]. Elderly who are allergic to nuts should read all food labels carefully and if the meals are not prepared by themselves, they should enquire about the ingredients in the meals [95]. Mycotoxins such as fumonisin B1, B2, and B3 are toxic substances produced by *Fusarium* moulds and have been found in nuts [96,97]. Antioxidants and phytochemicals in nuts have been shown to inhibit aflatoxin production [98]. Given that peanuts and tree nuts are not major components of the diet and rarely consumed in a large quantity, exposure to aflatoxins is likely to be low and hence detrimental health effects (if any) would be negligible. In addition, a recent study reported that the health benefits of nut consumption outweigh the potential risk from aflatoxin B1 exposure [99].

## 6. Future Research Directions

This review has revealed several areas that could be addressed in future research in order to gain a better understanding on the role of nuts in preventing and reversing malnutrition among elderly who are at risk of undernutrition:

• Numerous studies have investigated the effects of nut consumption on cardiovascular risk factors in healthy adults, overweight or obese individuals, etc. Very few studies were specifically designed to investigate the effects of nuts on health outcomes in the elderly. To our knowledge, no studies to date have examined the effects of nut consumption on nutritional status in elderly who suffer from or are at risk of malnutrition. Thus, randomised controlled trials are warranted to elucidate the underlying mechanisms and the potential role of nuts in improving nutritional status in the elderly.

• Given that elderly may have reduced appetite, it is important to ensure that foods they consume are resistant to monotony, so they will continue to consume it over a prolonged period. A few previous studies have investigated the effects of regular nut consumption on acceptability in adults and shown that nuts are resistant to monotony and no decline in liking was observed after daily consumption of nuts for up to twelve weeks. Future research should measure the acceptability of nuts in elderly who are at risk of undernutrition. A variety of nuts can be used to prevent sensory-specific satiety and promote nut intake.

• Future studies should investigate the timing and frequency of nut consumption in elderly in order to find out the ideal time to promote this healthful food. In addition, it would be interesting to examine the best way to incorporate nuts into the diet (e.g., as a meal, as part of a meal, as a snack or as part of a snack), without displacing other foods in their diet.

• Most nut studies carried out to date have used whole unsalted raw nuts. It would therefore be interesting to determine whether the health effects observed with raw nut consumption can be generalized to nuts with different physical forms (i.e., differing in the amount of chewing required) or preparation methods or flavours (i.e., roasting vs. frying; salted/honey roasted/chocolate-coated; low vs. high sodium content). In addition, future research is needed to determine the type and dose of nuts required to improve nutritional status of elderly at risk of undernutrition. 

## Figures and Tables

**Table 1:** The contribution of different nuts to the Recommended Daily Intake (RDI) or Recommended Dietary Allowance (RDA), and Adequate Intake (AI) from New Zealand/Australia and the USA.

		NZ/Australia RDI/AI				USA RDA/AI			
		Males		Females		Males		Females	
		51–70 years	>70 years	51–70 years	>70 years	51–70 years	>70 years	51–70 years	>70 years
Protein (g)	Amount per 30 g	% Daily Value							
		64 *	81 *	46 *	57 *	56 *	56 *	46 *	46 *
Almonds	6.35	9.9	7.8	13.8	11.1	11.3	11.3	13.8	13.8
Brazil nuts	4.30	6.7	5.3	9.3	7.5	7.7	7.7	9.3	9.3
Cashew nuts	5.30	8.3	6.5	11.5	9.3	9.5	9.5	11.5	11.5
Hazelnuts	4.49	7.0	5.5	9.8	7.9	8.0	8.0	9.8	9.8
Macadamia nuts	2.37	3.7	2.9	5.2	4.2	4.2	4.2	5.2	5.2
Peanuts	7.74	12.1	9.6	16.8	13.6	13.8	13.8	16.8	16.8
Pecans	2.75	4.3	3.4	6.0	4.8	4.9	4.9	6.0	6.0
Pine nuts	4.11	6.4	5.1	8.9	7.2	7.3	7.3	8.9	8.9
Pistachios	6.05	9.5	7.5	13.2	10.6	10.8	10.8	13.2	13.2
Walnuts	4.57	7.1	5.6	9.9	8.0	8.2	8.2	9.9	9.9
Range	2.37–7.74	3.7–12.1	2.9–9.6	5.2–16.8	4.2–13.6	4.2–13.8	4.2–13.8	5.2–16.8	5.2–16.8
Dietary fibre (g)	Amount per 30 g	% Daily Value							
		30 †	30 †	25 †	25 †	30 †	30 †	21 †	21 †
Almonds	3.75	12.5	12.5	15.0	15.0	12.5	12.5	17.9	17.9
Brazil nuts	2.25	7.5	7.5	9.0	9.0	7.5	7.5	10.7	10.7
Cashew nuts	0.99	3.3	3.3	4.0	4.0	3.3	3.3	4.7	4.7
Hazelnuts	2.91	9.7	9.7	11.6	11.6	9.7	9.7	13.9	13.9
Macadamia nuts	2.58	8.6	8.6	10.3	10.3	8.6	8.6	12.3	12.3
Peanuts	2.55	8.5	8.5	10.2	10.2	8.5	8.5	12.1	12.1
Pecans	2.88	9.6	9.6	11.5	11.5	9.6	9.6	13.7	13.7
Pine nuts	1.11	3.7	3.7	4.4	4.4	3.7	3.7	5.3	5.3
Pistachios	3.18	10.6	10.6	12.7	12.7	10.6	10.6	15.1	15.1
Walnuts	2.01	6.7	6.7	8.0	8.0	6.7	6.7	9.6	9.6
Range	0.99–3.75	3.3–12.5	3.3–12.5	4.0–15.0	4.0–15.0	3.3–12.5	3.3–12.5	4.7–17.9	4.7–17.9
α-Tocopherol (mg)	Amount per 30 g	% Daily Value							
		10 †	10 †	7 †	7 †	15 *	15 *	15 *	15 *
Almonds	7.69	76.9	76.9	109.9	109.9	51.3	51.3	51.3	51.3
Brazil nuts	1.70	17.0	17.0	24.3	24.3	11.3	11.3	11.3	11.3
Cashew nuts	0.90	9.0	9.0	12.9	12.9	6.0	6.0	6.0	6.0
Hazelnuts	5.51	55.1	55.1	78.7	78.7	36.7	36.7	36.7	36.7
Macadamia nuts	0.16	1.6	1.6	2.3	2.3	1.1	1.1	1.1	1.1
Peanuts	2.50	25.0	25.0	35.7	35.7	16.7	16.7	16.7	16.7
Pecans	0.42	4.2	4.2	6.0	6.0	2.8	2.8	2.8	2.8
Pine nuts	2.80	28.0	28.0	40.0	40.0	18.7	18.7	18.7	18.7
Pistachios	0.86	8.6	8.6	12.3	12.3	5.7	5.7	5.7	5.7
Walnuts	0.21	2.1	2.1	3.0	3.0	1.4	1.4	1.4	1.4
Range	0.16–7.69	1.6–76.9	1.6–76.9	2.3–109.9	2.3–109.9	1.1–51.3	1.1–51.3	1.1–51.3	1.1–51.3
Folate (µg)	Amount per 30 g	% Daily Value							
		400 *	400 *	400 *	400 *	400 *	400 *	400 *	400 *
Almonds	13.2	3.3	3.3	3.3	3.3	3.3	3.3	3.3	3.3
Brazil nuts	6.6	1.7	1.7	1.7	1.7	1.7	1.7	1.7	1.7
Cashew nuts	7.5	1.9	1.9	1.9	1.9	1.9	1.9	1.9	1.9
Hazelnuts	33.9	8.5	8.5	8.5	8.5	8.5	8.5	8.5	8.5
Macadamia nuts	3.3	0.8	0.8	0.8	0.8	0.8	0.8	0.8	0.8
Peanuts	72.0	18.0	18.0	18.0	18.0	18.0	18.0	18.0	18.0
Pecans	6.6	1.7	1.7	1.7	1.7	1.7	1.7	1.7	1.7
Pine nuts	10.2	2.6	2.6	2.6	2.6	2.6	2.6	2.6	2.6
Pistachios	15.3	3.8	3.8	3.8	3.8	3.8	3.8	3.8	3.8
Walnuts	29.4	7.4	7.4	7.4	7.4	7.4	7.4	7.4	7.4
Range	3.3–72.0	0.8–18.0	0.8–18.0	0.8–18.0	0.8–18.0	0.8–18.0	0.8–18.0	0.8–18.0	0.8–18.0
Calcium (mg)	Amount per 30 g	% Daily Value							
		1000 *	1300 *	1300 *	1300 *	1000 *	1200 *	1200 *	1200 *
Almonds	80.7	8.1	6.2	6.2	6.2	8.1	6.7	6.7	6.7
Brazil nuts	48.0	4.8	3.7	3.7	3.7	4.8	4.0	4.0	4.0
Cashew nuts	11.1	1.1	0.9	0.9	0.9	1.1	0.9	0.9	0.9
Hazelnuts	34.2	3.4	2.6	2.6	2.6	3.4	2.9	2.9	2.9
Macadamia nuts	25.5	2.6	2.0	2.0	2.0	2.6	2.1	2.1	2.1
Peanuts	27.7	2.8	2.1	2.1	2.1	2.8	2.3	2.3	2.3
Pecans	21.0	2.1	1.6	1.6	1.6	2.1	1.8	1.8	1.8
Pine nuts	4.8	0.5	0.4	0.4	0.4	0.5	0.4	0.4	0.4
Pistachios	31.5	3.2	2.4	2.4	2.4	3.2	2.6	2.6	2.6
Walnuts	29.0	2.9	2.2	2.2	2.2	2.9	2.4	2.4	2.4
Range	4.8–80.7	0.5–8.1	0.4–6.2	0.4–6.2	0.4–6.2	0.5–8.1	0.4–6.7	0.4–6.7	0.4–6.7
Copper (µg)	Amount per 30 g	% Daily Value							
		1700 †	1700 †	1200 †	1200 †	900 *	900 *	900 *	900 *
Almonds	309	18.2	18.2	25.8	25.8	34.3	34.3	34.3	34.3
Brazil nuts	523	30.8	30.8	43.6	43.6	58.1	58.1	58.1	58.1
Cashew nuts	659	38.8	38.8	54.9	54.9	73.2	73.2	73.2	73.2
Hazelnuts	518	30.5	30.5	43.2	43.2	57.6	57.6	57.6	57.6
Macadamia nuts	227	13.4	13.4	18.9	18.9	25.2	25.2	25.2	25.2
Peanuts	343	20.2	20.2	28.6	28.6	38.1	38.1	38.1	38.1
Pecans	360	21.2	21.2	30.0	30.0	40.0	40.0	40.0	40.0
Pine nuts	397	23.4	23.4	33.1	33.1	44.1	44.1	44.1	44.1
Pistachios	390	22.9	22.9	32.5	32.5	43.3	43.3	43.3	43.3
Walnuts	476	28.0	28.0	39.7	39.7	52.9	52.9	52.9	52.9
Range	277–659	13.4–38.8	13.4–38.8	18.9–54.9	18.9–54.9	25.2–73.2	25.2–73.2	25.2–73.2	25.2–73.2
Iron (mg)	Amount per 30 g	% Daily Value							
		8 *	8 *	8 *	8 *	8 *	8 *	8 *	8 *
Almonds	1.11	13.9	13.9	13.9	13.9	13.9	13.9	13.9	13.9
Brazil nuts	0.73	9.1	9.1	9.1	9.1	9.1	9.1	9.1	9.1
Cashew nuts	2.00	25.0	25.0	25.0	25.0	25.0	25.0	25.0	25.0
Hazelnuts	1.41	17.6	17.6	17.6	17.6	17.6	17.6	17.6	17.6
Macadamia nuts	1.11	13.9	13.9	13.9	13.9	13.9	13.9	13.9	13.9
Peanuts	1.37	17.1	17.1	17.1	17.1	17.1	17.1	17.1	17.1
Pecans	0.76	9.5	9.5	9.5	9.5	9.5	9.5	9.5	9.5
Pine nuts	1.55	19.4	19.4	19.4	19.4	19.4	19.4	19.4	19.4
Pistachios	1.18	14.8	14.8	14.8	14.8	14.8	14.8	14.8	14.8
Walnuts	0.87	10.9	10.9	10.9	10.9	10.9	10.9	10.9	10.9
Range	0.73–2.00	9.1–25.0	9.1–25.0	9.1–25.0	9.1–25.0	9.1–25.0	9.1–25.0	9.1–25.0	9.1–25.0
Magnesium (mg)	Amount per 30 g	% Daily Value							
		420 *	420 *	320 *	320 *	420 *	420 *	320 *	320 *
Almonds	81	19.3	19.3	25.3	25.3	19.3	19.3	25.3	25.3
Brazil nuts	113	26.9	26.9	35.3	35.3	26.9	26.9	35.3	35.3
Cashew nuts	88	21.0	21.0	27.5	27.5	21.0	21.0	27.5	27.5
Hazelnuts	49	11.7	11.7	15.3	15.3	11.7	11.7	15.3	15.3
Macadamia nuts	39	9.3	9.3	12.2	12.2	9.3	9.3	12.2	12.2
Peanuts	50	11.9	11.9	15.6	15.6	11.9	11.9	15.6	15.6
Pecans	36	8.6	8.6	11.3	11.3	8.6	8.6	11.3	11.3
Pine nuts	75	17.9	17.9	23.4	23.4	17.9	17.9	23.4	23.4
Pistachios	36	8.6	8.6	11.3	11.3	8.6	8.6	11.3	11.3
Walnuts	47	11.2	11.2	14.7	14.7	11.2	11.2	14.7	14.7
Range	36–113	8.6–26.9	8.6–26.9	11.3–35.3	11.3–35.3	8.6–26.9	8.6–26.9	11.3–35.3	11.3–35.3
Phosphorous (mg)	Amount per 30 g	% Daily Value							
		1000 *	1000 *	1000 *	1000 *	700 *	700 *	700 *	700 *
Almonds	144	14.4	14.4	14.4	14.4	20.6	20.6	20.6	20.6
Brazil nuts	218	21.8	21.8	21.8	21.8	31.1	31.1	31.1	31.1
Cashew nuts	178	17.8	17.8	17.8	17.8	25.4	25.4	25.4	25.4
Hazelnuts	87	8.7	8.7	8.7	8.7	12.4	12.4	12.4	12.4
Macadamia nuts	56	5.6	5.6	5.6	5.6	8.0	8.0	8.0	8.0
Peanuts	113	11.3	11.3	11.3	11.3	16.1	16.1	16.1	16.1
Pecans	83	8.3	8.3	8.3	8.3	11.9	11.9	11.9	11.9
Pine nuts	173	17.3	17.3	17.3	17.3	24.7	24.7	24.7	24.7
Pistachios	147	14.7	14.7	14.7	14.7	21.0	21.0	21.0	21.0
Walnuts	104	10.4	10.4	10.4	10.4	14.9	14.9	14.9	14.9
Range	56–218	5.6–21.8	5.6–21.8	5.6–21.8	5.6–21.8	8.0–31.1	8.0–31.1	8.0–31.1	8.0–31.1
Potassium (mg)	Amount per 30 g	% Daily Value							
		3800 †	3800 †	2800 †	2800 †	4700 †	4700 †	4700 †	4700 †
Almonds	220	5.8	5.8	7.9	7.9	4.7	4.7	4.7	4.7
Brazil nuts	198	5.2	5.2	7.1	7.1	4.2	4.2	4.2	4.2
Cashew nuts	198	5.2	5.2	7.1	7.1	4.2	4.2	4.2	4.2
Hazelnuts	204	5.4	5.4	7.3	7.3	4.3	4.3	4.3	4.3
Macadamia nuts	110	2.9	2.9	3.9	3.9	2.3	2.3	2.3	2.3
Peanuts	212	5.6	5.6	7.6	7.6	4.5	4.5	4.5	4.5
Pecans	123	3.2	3.2	4.4	4.4	2.6	2.6	2.6	2.6
Pine nuts	179	4.7	4.7	6.4	6.4	3.8	3.8	3.8	3.8
Pistachios	308	8.1	8.1	11.0	11.0	6.6	6.6	6.6	6.6
Walnuts	132	3.5	3.5	4.7	4.7	2.8	2.8	2.8	2.8
Range	110–308	2.9–8.1	2.9–8.1	3.9–11.0	3.9–11.0	2.3–6.6	2.3–6.6	2.3–6.6	2.3–6.6
Selenium (µg)	Amount per 30 g	% Daily Value							
		70 *	70 *	60 *	60 *	55 *	55 *	55 *	55 *
Almonds	1.23	1.8	1.8	2.1	2.1	2.2	2.2	2.2	2.2
Brazil nuts	575	821	821	958	958	1045	1045	1045	1045
Cashew nuts	5.97	8.5	8.5	10.0	10.0	10.9	10.9	10.9	10.9
Hazelnuts	0.72	1.0	1.0	1.2	1.2	1.3	1.3	1.3	1.3
Macadamia nuts	1.08	1.5	1.5	1.8	1.8	2.0	2.0	2.0	2.0
Peanuts	2.16	3.1	3.1	3.6	3.6	3.9	3.9	3.9	3.9
Pecans	1.14	1.6	1.6	1.9	1.9	2.1	2.1	2.1	2.1
Pine nuts	0.21	0.3	0.3	0.4	0.4	0.4	0.4	0.4	0.4
Pistachios	2.1	3.0	3.0	3.5	3.5	3.8	3.8	3.8	3.8
Walnuts	1.47	2.1	2.1	2.5	2.5	2.7	2.7	2.7	2.7
Range	0.21–575	0.3–821	0.3–821	0.4–958	0.4–958	0.4–1045	0.4–1045	0.4–1045	0.4–1045
Sodium (mg)	Amount per 30 g	% Daily Value							
		460–920 †	460–920 †	460–920 †	460–920 †	1300 †	1200 †	1300 †	1200 †
Almonds	0.3	0.03–0.07	0.03–0.07	0.03–0.07	0.03–0.07	0.02	0.03	0.02	0.03
Brazil nuts	0.9	0.10–0.20	0.10–0.20	0.10–0.20	0.10–0.20	0.07	0.08	0.07	0.08
Cashew nuts	3.6	0.39–0.78	0.39–0.78	0.39–0.78	0.39–0.78	0.28	0.30	0.28	0.30
Hazelnuts	0.0	0.00	0.00	0.00	0.00	0.00	0.00	0.00	0.00
Macadamia nuts	1.5	0.16–0.33	0.16–0.33	0.16–0.33	0.16–0.33	0.12	0.13	0.12	0.13
Peanuts	5.4	0.59–1.17	0.59–1.17	0.59–1.17	0.59–1.17	0.42	0.45	0.42	0.45
Pecans	0.0	0.00	0.00	0.00	0.00	0.00	0.00	0.00	0.00
Pine nuts	0.6	0.07–0.13	0.07–0.13	0.07–0.13	0.07–0.13	0.05	0.05	0.05	0.05
Pistachios	0.3	0.03–0.07	0.03–0.07	0.03–0.07	0.03–0.07	0.02	0.03	0.02	0.03
Walnuts	0.6	0.07–0.13	0.07–0.13	0.07–0.13	0.07–0.13	0.05	0.05	0.05	0.05
Range	0.0–5.4	0.00–1.17	0.00–1.17	0.00–1.17	0.00–1.17	0.00–0.42	0.00–0.45	0.00–0.42	0.00–0.45
Zinc (mg)	Amount per 30 g	% Daily Value							
		12 *	12 *	6.5 *	6.5 *	11 *	11 *	8 *	8 *
Almonds	0.94	7.8	7.8	14.5	14.5	8.5	8.5	11.8	11.8
Brazil nuts	1.28	10.7	10.7	19.7	19.7	11.6	11.6	16.0	16.0
Cashew nuts	1.73	14.4	14.4	26.6	26.6	15.7	15.7	21.6	21.6
Hazelnuts	0.74	6.2	6.2	11.4	11.4	6.7	6.7	9.3	9.3
Macadamia nuts	0.39	3.3	3.3	6.0	6.0	3.5	3.5	4.9	4.9
Peanuts	0.98	8.2	8.2	15.1	15.1	8.9	8.9	12.3	12.3
Pecans	1.36	11.3	11.3	20.9	20.9	12.4	12.4	17.0	17.0
Pine nuts	1.94	16.2	16.2	29.8	29.8	17.6	17.6	24.3	24.3
Pistachios	0.66	5.5	5.5	10.2	10.2	6.0	6.0	8.3	8.3
Walnuts	0.93	7.8	7.8	14.3	14.3	8.5	8.5	11.6	11.6
Range	0.39–1.94	3.3–16.2	3.3–16.2	6.0–29.8	6.0–29.8	3.5–17.6	3.5–17.6	4.9–24.3	4.9–24.3

* Value is an RDA/RDI; † Value is an AI; All values are sourced from the US Department of Agriculture National Nutrient Database for Standard Reference Release 28 (slightly revised May 2016) Software v.3.8.6.5 [71].

## References

[1] United Nations World population ageing 2017: Highlights. https://www.google.com.tw/url?sa=t&rct=j&q=&esrc=s&source=web&cd=4&ved=2ahUKEwjypbvvyeHdAhVGIIgKHQ51CnYQFjADegQICRAC&url=http%3A%2F%2Fwww.un.org%2Fen%2Fdevelopment%2Fdesa%2Fpopulation%2Fpublications%2Fpdf%2Fageing%2FWPA2017_Highlights.pdf&usg=AOvVaw29cbhXyZhSy_ipICc-g9vB.

[2] Wu L.L., Cheung K.Y., Lam P.Y.P., Gao X. (2017). Oral health indicators for risk of malnutrition in elders. J. Nutr. Health Aging.

[3] Morley J.E. (1997). Anorexia of aging: Physiologic and pathologic. Am. J. Clin. Nutr..

[4] Landi F., Calvani R., Tosato M., Martone A., Ortolani E., Savera G., Sisto A., Marzetti E. (2016). Anorexia of aging: Risk factors, consequences, and potential treatments. Nutrients.

[5] Abizanda P., Sinclair A., Barcons N., Lizán L., Rodríguez-Mañas L. (2016). Costs of malnutrition in institutionalized and community-dwelling older adults: a systematic review. J. Am. Méd. Dir. Assoc..

[6] Freijer K., Tan S.S., Koopmanschap M.A., Meijers J.M.M., Halfens R.J.G., Nuijten M.J.C. (2013). The economic costs of disease related malnutrition. Clin. Nutr..

[7] Muscaritoli M., Molfino A. (2013). Malnutrition: The hidden killer in healthcare systems. BMJ.

[8] Webb P., Stordalen G.A., Singh S., Wijesinha-Bettoni R., Shetty P., Lartey A. (2018). Hunger and malnutrition in the 21st century. BMJ.

[9] World Health Organization The double burden of malnutrition: Policy brief. http://www.who.int/nutrition/publications/doubleburdenmalnutrition-policybrief/en/.

[10] Winter J., Flanagan D., McNaughton S.A., Nowson C. (2013). Nutrition screening of older people in a community general practice, using the mna-sf. J. Nutr. Health Aging.

[11] Cereda E., Pedrolli C., Klersy C., Bonardi C., Quarleri L., Cappello S., Turri A., Rondanelli M., Caccialanza R. (2016). Nutritional status in older persons according to healthcare setting: A systematic review and meta-analysis of prevalence data using mna. Clin. Nutr..

[12] John B.K., Bullock M., Brenner L., McGaw C., Scolapio J.S. (2013). Nutrition in the elderly. Frequently asked questions. Am. J. Gastroenterol..

[13] Doets E.L., Kremer S. (2016). The silver sensory experience—a review of senior consumers’ food perception, liking and intake. Food Qual. Prefer..

[14] Rothenberg E., Wendin K., Chen J., Rosenthal A. (2015). 7-texture modification of food for elderly people. Modifying Food Texture.

[15] Quandt S.A., Chen H., Bell R.A., Savoca M.R., Anderson A.M., Leng X., Kohrman T., Gilbert G.H., Arcury T.A. (2010). Food avoidance and food modification practices of older rural adults: Association with oral health status and implications for service provision. Gerontologist.

[16] Bernstein M., Munoz N. (2012). Position of the academy of nutrition and dietetics: Food and nutrition for older adults: Promoting health and wellness. J. Acad. Nutr. Diet..

[17] Wu S.-J., Ya-Hui C., Wei I.-L., Kao M.-D., Yi-Chin L., Pan W.-H. (2005). Intake levels and major food sources of energy and nutrients in the taiwanese elderly. Asia Pac. J. Clin. Nutr..

[18] World Health Organization (2017). Integrated care for older people: Guidelines on community-level interventions to manage declines in intrinsic capacity.

[19] Lamuel-Raventos R.M., Onge M.-P.S. (2017). Prebiotic nut compounds and human microbiota. Crit. Rev. Food Sci. Nutr..

[20] Alasalvar C., Bolling B.W. (2015). Review of nut phytochemicals, fat-soluble bioactives, antioxidant components and health effects. Br. J. Nutr..

[21] Tan S.-Y., Dhillon J., Mattes R.D. (2014). A review of the effects of nuts on appetite, food intake, metabolism, and body weight. Am. J. Clin. Nutr..

[22] Ledikwe J.H., Blanck H.M., Kettel Khan L., Serdula M.K., Seymour J.D., Tohill B.C., Rolls B.J. (2006). Dietary energy density is associated with energy intake and weight status in us adults. Am. J. Clin. Nutr..

[23] Vernarelli J.A., Mitchell D.C., Hartman T.J., Rolls B.J. (2011). Dietary energy density is associated with body weight status and vegetable intake in U.S. Children. J. Nutr..

[24] Wansink B., Painter J.E., North J. (2005). Bottomless bowls: Why visual cues of portion size may influence intake. Obes. Res..

[25] Tey S.L., Chia E.M.E., Forde C.G. (2016). Impact of dose-response calorie reduction or supplementation of a covertly manipulated lunchtime meal on energy compensation. Physiol. Behav..

[26] McCrickerd K., Salleh N., Forde C. (2016). Removing energy from a beverage influences later food intake more than the same energy addition. Appetite.

[27] Rolls B.J., Bell E.A. (1999). Intake of fat and carbohydrate: Role of energy density. Eur. J. Clin. Nutr..

[28] Lawrence M., Wingrove K., Naude C., Durao S. (2016). Evidence synthesis and translation for nutrition interventions to combat micronutrient deficiencies with particular focus on food fortification. Nutrients.

[29] Mills S., Wilcox C., Ibrahim K., Roberts H. (2018). Can fortified foods and snacks increase the energy and protein intake of hospitalised older patients? A systematic review. J. Hum. Nutr. Diet..

[30] Trabal J., Farran-Codina A. (2015). Effects of dietary enrichment with conventional foods on energy and protein intake in older adults: A systematic review. Nutr. Rev..

[31] Guasch-Ferré M., Liu X., Malik V.S., Sun Q., Willett W.C., Manson J.E., Rexrode K.M., Li Y., Hu F.B., Bhupathiraju S.N. (2017). Nut consumption and risk of cardiovascular disease. J. Am. Coll. Cardiol..

[32] Barbour J.A., Howe P.R., Buckley J.D., Bryan J., Coates A.M. (2014). Nut consumption for vascular health and cognitive function. Nutr. Res. Rev..

[33] Saaka M., Osman S.M., Amponsem A., Ziem J.B., Abdul-Mumin A., Akanbong P., Yirkyio E., Yakubu E., Ervin S. (2015). Treatment outcome of severe acute malnutrition cases at the tamale teaching hospital. J. Nutr. Metab..

[34] Defourny I., Minetti A., Harczi G., Doyon S., Shepherd S., Tectonidis M., Bradol J.-H., Golden M. (2009). A large-scale distribution of milk-based fortified spreads: Evidence for a new approach in regions with high burden of acute malnutrition. PloS one.

[35] Yebyo H.G., Kendall C., Nigusse D., Lemma W. (2013). Outpatient therapeutic feeding program outcomes and determinants in treatment of severe acute malnutrition in tigray, northern ethiopia: A retrospective cohort study. Plos one.

[36] Powley T.L., Phillips R.J. (2004). Gastric satiation is volumetric, intestinal satiation is nutritive. Physiol. Behav..

[37] Mattes R.D., Hollis J., Hayes D., Stunkard A.J. (2005). Appetite: Measurement and manipulation misgivings. J. Am. Diet. Assoc..

[38] Dhillon J., Running C.A., Tucker R.M., Mattes R.D. (2016). Effects of food form on appetite and energy balance. Food Qual. Prefer..

[39] Ello-Martin J.A., Ledikwe J.H., Rolls B.J. (2005). The influence of food portion size and energy density on energy intake: Implications for weight management. Am. J. Clin. Nutr..

[40] Dhillon J., Craig B.A., Leidy H.J., Amankwaah A.F., Anguah K.O.-B., Jacobs A., Jones B.L., Jones J.B., Keeler C.L., Keller C.E. (2016). The effects of increased protein intake on fullness: A meta-analysis and its limitations. J. Acad. Nutr. Diet..

[41] Clark M.J., Slavin J.L. (2013). The effect of fiber on satiety and food intake: A systematic review. J. Am. Coll. Nutr..

[42] de Graaf C. (2012). Texture and satiation: The role of oro-sensory exposure time. Physiol. Behav..

[43] Fraser G.E., Bennett H.W., Jaceldo K.B., Sabate J. (2002). Effect on body weight of a free 76 kilojoule (320 calorie) daily supplement of almonds for six months. J. Am. Coll. Nutr..

[44] Kirkmeyer S.V., Mattes R.D. (2000). Effects of food attributes on hunger and food intake. Int. J. Obes. Relat. Metab. Disord..

[45] Hollis J., Mattes R. (2007). Effect of chronic consumption of almonds on body weight in healthy humans. Br. J. Nutr..

[46] Alper C.M., Mattes R.D. (2002). Effects of chronic peanut consumption on energy balance and hedonics. Int. J. Obes. Relat. Metab. Disord..

[47] Ellis P.R., Kendall C.W.C., Ren Y.L., Parker C., Pacy J.F., Waldron K.W., Jenkins D.J.A. (2004). Role of cell walls in the bioaccessibility of lipids in almond seeds. Am. J. Clin. Nutr..

[48] Novotny J.A., Gebauer S.K., Baer D.J. (2012). Discrepancy between the atwater factor predicted and empirically measured energy values of almonds in human diets. Am. J. Clin. Nutr..

[49] Baer D.J., Gebauer S.K., Novotny J.A. (2012). Measured energy value of pistachios in the human diet. Br. J. Nutr..

[50] Baer D.J., Gebauer S.K., Novotny J.A. (2015). Walnuts consumed by healthy adults provide less available energy than predicted by the atwater factors. J. Nutr..

[51] Grundy M.M.L., Grassby T., Mandalari G., Waldron K.W., Butterworth P.J., Berry S.E.E., Ellis P.R. (2015). Effect of mastication on lipid bioaccessibility of almonds in a randomized human study and its implications for digestion kinetics, metabolizable energy, and postprandial lipemia. Am. J. Clin. Nutr..

[52] Frecka J., Hollis J., Mattes R. (2008). Effects of appetite, BMI, food form and flavor on mastication: Almonds as a test food. Eur. J. Clin. Nutr..

[53] Gebauer S.K., Novotny J.A., Bornhorst G.M., Baer D.J. (2016). Food processing and structure impact the metabolizable energy of almonds. Food Funct..

[54] McKiernan F., Mattes R.D. (2010). Effects of peanut processing on masticatory performance during variable appetitive states. J. Nutr. Metab..

[55] Traoret C., Lokko P., Cruz A., Oliveira C., Costa N., Bressan J., Alfenas R., Mattes R. (2008). Peanut digestion and energy balance. Int. J. Obes..

[56] McKiernan F., Lokko P., Kuevi A., Sales R.L., Costa N.M., Bressan J., Alfenas R.C., Mattes R.D. (2010). Effects of peanut processing on body weight and fasting plasma lipids. Br. J. Nutr..

[57] Pasman W.J., Heimerikx J., Rubingh C.M., van den Berg R., O’Shea M., Gambelli L., Hendriks H.F.J., Einerhand A.W.C., Scott C., Keizer H.G. (2008). The effect of korean pine nut oil on in vitro cck release, on appetite sensations and on gut hormones in post-menopausal overweight women. Lipids Health Dis..

[58] Lauque S., Arnaud-Battandier F., Mansourian R., Guigoz Y., Paintin M., Nourhashemi F., Vellas B. (2000). Protein-energy oral supplementation in malnourished nursing-home residents. A controlled trial. Age Ageing.

[59] Nieuwenhuizen W.F., Weenen H., Rigby P., Hetherington M.M. (2010). Older adults and patients in need of nutritional support: Review of current treatment options and factors influencing nutritional intake. Clin. Nutr..

[60] Hubbard G.P., Elia M., Holdoway A., Stratton R.J. (2012). A systematic review of compliance to oral nutritional supplements. Clin. Nutr..

[61] Marcenes W., Steele J.G., Sheiham A., Walls A.W.G. (2003). The relationship between dental status, food selection, nutrient intake, nutritional status, and body mass index in older people. Cad. Saude Publica.

[62] Rolls B.J. (1986). Sensory-specific satiety. Nutr. Rev..

[63] Tey S.L., Delahunty C., Gray A., Chisholm A., Brown R.C. (2015). Effects of regular consumption of different forms of almonds and hazelnuts on acceptance and blood lipids. Eur. J. Nutr..

[64] Jones J.B., Provost M., Keaver L., Breen C., Ludy M.-J., Mattes R.D. (2014). A randomized trial on the effects of flavorings on the health benefits of daily peanut consumption. Am. J. Clin. Nutr..

[65] Tey S.L., Brown R., Chisholm A., Delahunty C., Gray A., Williams S. (2011). Effects of different forms of hazelnuts on blood lipids and α-tocopherol concentrations in mildly hypercholesterolemic individuals. Eur. J. Clin. Nutr..

[66] Tey S.L., Brown R., Chisholm A., Gray A., Williams S., Delahunty C. (2011). Current guidelines for nut consumption are achievable and sustainable: A hazelnut intervention. Br. J. Nutr..

[67] Stroehla B.C., Malcoe L.H., Velie E.M. (2005). Dietary sources of nutrients among rural native american and white children. J. Am. Diet. Assoc..

[68] Nicklas T.A., Demory-Luce D., Yang S.J., Baranowski T., Zakeri I., Berenson G. (2004). Children’s food consumption patterns have changed over two decades (1973–1994): The bogalusa heart study. J. Am. Diet. Assoc..

[69] Zizza C., Arsiwalla D.D., Ellison K.J. (2010). Contribution of snacking to older adults’ vitamin, carotenoid, and mineral intakes. J. Am. Diet. Assoc..

[70] Ros E. (2010). Health benefits of nut consumption. Nutrients.

[71] USDA National Nutrient Database for Standard Reference, Release 28. https://www.google.com.tw/url?sa=t&rct=j&q=&esrc=s&source=web&cd=16&ved=2ahUKEwiayNns6uTdAhXH7GEKHZYHBtw4ChAWMAV6BAgBEAI&url=https%3A%2F%2Fods.od.nih.gov%2Fpubs%2Fusdandb%2FCalcium-Content.pdf&usg=AOvVaw2csgADPanW96U7T_06Qu_G.

[72] Brown R.C., Tey S.L., Gray A.R., Chisholm A., Smith C., Fleming E., Parnell W. (2016). Nut consumption is associated with better nutrient intakes: Results from the 2008/09 New Zealand adult nutrition survey. Br. J. Nutr..

[73] King J.C., Blumberg J., Ingwersen L., Jenab M., Tucker K.L. (2008). Tree nuts and peanuts as components of a healthy diet. J. Nutr..

[74] O’Neil C.E., Keast D.R., Fulgoni V.L., Nicklas T.A. (2010). Tree nut consumption improves nutrient intake and diet quality in us adults: An analysis of national health and nutrition examination survey (nhanes) 1999–2004. Asia Pac. J. Clin. Nutr..

[75] O’Neil C.E., Keast D.R., Nicklas T.A., Fulgoni V.L. (2012). Out-of-hand nut consumption is associated with improved nutrient intake and health risk markers in us children and adults: National health and nutrition examination survey 1999–2004. Nutr. Res..

[76] O’Neil C.E., Nicklas T.A., Fulgoni III V.L. (2015). Tree nut consumption is associated with better nutrient adequacy and diet quality in adults: National health and nutrition examination survey 2005–2010. Nutrients.

[77] Griel A.E., Eissenstat B., Juturu V., Hsieh G., Kris-Etherton P.M. (2004). Improved diet quality with peanut consumption. J. Am. Coll. Nutr..

[78] Bitok E., Jaceldo-Siegl K., Rajaram S., Serra-Mir M., Roth I., Feitas-Simoes T., Ros E., Sabaté J. (2017). Favourable nutrient intake and displacement with long-term walnut supplementation among elderly: Results of a randomised trial. Br. J. Nutr..

[79] Jaceldo-Siegl K., Joan S., Rajaram S., Fraser G.E. (2004). Long-term almond supplementation without advice on food replacement induces favourable nutrient modifications to the habitual diets of free-living individuals. Br. J. Nutr..

[80] Tey S.L., Brown R., Gray A., Chisholm A., Delahunty C. (2011). Nuts improve diet quality compared to other energy-dense snacks while maintaining body weight. J. Nutr. Metab..

[81] Tey S.L., Gray A.R., Chisholm A.W., Delahunty C.M., Brown R.C. (2013). The dose of hazelnuts influences acceptance and diet quality but not inflammatory markers and body composition in overweight and obese individuals. J. Nutr..

[82] Harland B.F., Smikle-Williams S., Oberleas D. (2004). High performance liquid chromatography analysis of phytate (ip6) in selected foods. J. Food Compost. Anal..

[83] Hurrell (2004). Phytic acid degradation as a means of improving iron absorption. Int. J. Vitam. Nutr. Res..

[84] Venkatachalam M., Sathe S.K. (2006). Chemical composition of selected edible nut seeds. J. Agric. Food Chem..

[85] International Zinc Nutrition Consultative Group (2004). International zinc nutrition consultative group (izincg) technical document# 1. Assessment of the risk of zinc deficiency in populations and options for its control. Food Nutr. Bull..

[86] Gupta R.K., Gangoliya S.S., Singh N.K. (2015). Reduction of phytic acid and enhancement of bioavailable micronutrients in food grains. J. Food Sci. Technol..

[87] Lestienne I., Mouquet-Rivier C., Icard-Vernière C., Rochette I., Trèche S. (2005). The effects of soaking of whole, dehulled and ground millet and soybean seeds on phytate degradation and phy/fe and phy/zn molar ratios. Int. J. Food Sci. Tech..

[88] Hotz C., Gibson R.S. (2001). Assessment of home-based processing methods to reduce the phytate content and phytate/zinc molar ratio of white maize (zea mays). J. Agric. Food Chem..

[89] Lin L., Giam X.Y., Foo X.M., Yeo H.L., Koh J.L., Sa’Aban N.H.B., Loke W.M. (2017). Effects of pre-germination treatment on the phytate and phenolic contents of almond nuts. J. Nuts.

[90] Taylor H., Webster K., Gray A.R., Tey S.L., Chisholm A., Bailey K., Kumari S., Brown R.C. (2017). The effects of ‘activating’ almonds on consumer acceptance and gastrointestinal tolerance. Eur. J. Nutr..

[91] Yong L.C., Gray A.R., Chisholm A., Leong S.L., Tey S.L., Brown R.C. (2017). Barriers to and facilitators and perceptions of nut consumption among the general population in New Zealand. Public Health Nutr..

[92] Brown R.C., Yong L.C., Gray A.R., Tey S.L., Chisholm A., Leong S.L. (2017). Perceptions and knowledge of nuts amongst health professionals in New Zealand. Nutrients.

[93] Brough H., Turner P., Wright T., Fox A., Taylor S., Warner J., Lack G. (2015). Dietary management of peanut and tree nut allergy: What exactly should patients avoid?. Clin. Exp. Allergy.

[94] Rona R.J., Keil T., Summers C., Gislason D., Zuidmeer L., Sodergren E., Sigurdardottir S.T., Lindner T., Goldhahn K., Dahlstrom J. (2007). The prevalence of food allergy: A meta-analysis. J. Allergy Clin. Immunol..

[95] Davis P.A., Jenab M., Vanden Heuvel J.P., Furlong T., Taylor S. (2008). Tree nut and peanut consumption in relation to chronic and metabolic diseases including allergy. J. Nutr..

[96] van Egmond H.P., Schothorst R.C., Jonker M.A. (2007). Regulations relating to mycotoxins in food: Perspectives in a global and european context. Anal. Bioanal. Chem..

[97] Edlayne G., Simone A., Felicio J.D. (2009). Chemical and biological approaches for mycotoxin control: A review. Recent Pat. Food Nutr. Agric..

[98] Alasalvar C., Shahidi F., Alasalvar C., Shahidi F. (2008). Tree nuts: Composition, phytochemicals, and health effects: An overview. Tree nuts: Composition, phytochemicals, and health effects.

[99] Eneroth H., Wallin S., Leander K., Nilsson Sommar J., Åkesson A. (2017). Risks and benefits of increased nut consumption: Cardiovascular health benefits outweigh the burden of carcinogenic effects attributed to aflatoxin b1 exposure. Nutrients.

